# COVID-19 Vaccination in Patients with Chronic Liver Disease

**DOI:** 10.3390/v14122778

**Published:** 2022-12-13

**Authors:** Georgios Schinas, Eleni Polyzou, Fevronia Mitropetrou, Aristotelis Pazionis, Charalambos Gogos, Christos Triantos, Karolina Akinosoglou

**Affiliations:** 1 Department of Internal Medicine, University General Hospital of Patras, 26504 Rio, Greece; 2 Department of Medicine, University of Patras, 26504 Rio, Greece; 3 Division of Gastroenterology, Department of Internal Medicine, University General Hospital of Patras, 26504 Rio, Greece

**Keywords:** COVID-19 vaccines, mRNA vaccine, chronic liver disease, immunization, SARS-CoV-2

## Abstract

Vaccination against SARS-CoV-2 has become a central public health issue, primarily for vulnerable populations such as individuals with Chronic Liver Disease (CLD). Increased COVID-19-related mortality and disease severity has been noted in this subgroup of patients. Severe COVID-19 tends to further deregulate liver function in patients with chronic liver failure or cirrhosis and even reactivate hepatitis in people living with HBV or HCV. In addition, impaired hepatic function leads to several limitations in possible therapeutic interventions. Chronic hepatic dysregulation, along with the underlying cirrhosis-associated immune dysfunction (CAID), leads to a decreased immune response to vaccination that, in turn, may result in reduced efficacy rates and lowered lasting protection. According to current guidelines, timely vaccination and frequent booster shot administration are deemed necessary in this context. Vaccination-related adverse events are mostly mild in nature and similar to those reported in the general population, whereas the incidence of liver injury following vaccination is relatively rare. We aimed to review available evidence and recommendations associated with COVID-19 vaccination in patients with chronic liver disease, and provide insight to current issues and future directions.

## 1. Introduction

Severe Acute Respiratory Syndrome Coronavirus 2, most commonly known as SARS-CoV-2 is an RNA virus first detected in Wuhan, China, on December 2019 that has caused global mayhem with its spread that led to the COVID-19 pandemic. In the following 3 years, the coronavirus disease resulted in significant mortality and morbidity, especially in people with chronic conditions and multiple comorbidities [1]. The scientific community proceeded in an exceptional fashion to develop, within a short period of time, a number of highly effective and safe vaccines in order to save lives and shield health systems worldwide. These vaccines have become widely accessible to the global community, with around 4.94 billion people having successfully completed the initial vaccination protocol, as of 12 September 2022 [2]. People with chronic liver disease (CLD) were among the first to be advised to get vaccinated due to their increased risk for adverse outcomes should they contract the virus. Immune dysfunction and chronic physiologic stress constitute them one of the most vulnerable patient groups. Living in an era of immense CLD burden, with more than 112 million people living with cirrhosis worldwide and 2 million deaths caused annually by hepatocellular carcinoma and/or hepatic decompensation [3,4], the significance of conveying timely and accurate information to this patient population regarding vaccination against SARS-CoV-2 becomes evident. We aimed to review available evidence and recommendations associated with COVID-19 vaccination in patients with chronic liver disease, and provide insight to current issues and future directions.

## 2. CLD and COVID-19

COVID-19 is primarily regarded as a respiratory tract infection by patients and physicians alike. However, the systemic distribution of viral particles and the presence of their ligand, including—but not limited to—ACE2 receptor, throughout the body, makes the scene for a systematic disease, that may affect all organ systems [5,6]. Even though, in this context, liver involvement has long been debated, recent multimodal evidence has come to confirm liver tropism of the virus and a molecular signature, featuring significant upregulation of IFN responses, JAK-STAT signaling and liver-specific metabolic modulation [7]. This is exemplified by the fact that elevation of liver enzymes seems to correlate with the disease severity and mortality in COVID-19 [8].

Pre-existing liver disease influences the outcome, as well as, the severity of SARS-CoV-2 infection [9,10,11]. CLD pertains to increased mortality risk (RR 2.8, 95% CI 1.9–4.0). Cirrhosis, in particular, increases the risk of death by COVID-19 about five times (RR 4.6, 95% CI 2.6–8.3) [12]. COVID-19-related mortality ranges from 22% in Child–Pugh A to 39% in Child–Pugh B and reached 54% in Child–Pugh C classified patients [13]. 

Although, mostly associated with bacterial insult, viral infections have been described to cause acute-on-chronic liver failure [14]. COVID-19 in patients with established cirrhosis appears to cause further liver dysfunction with the rate of acute hepatic decompensation in these individuals being noticeably high (46%) [13]. Up to 80% of those will eventually require intensive care unit (ICU) support. In addition, people living with Hepatitis B (HBV) and C (HCV) may experience reactivation of hepatitis, either due to COVID-19 per se or due to treatment [15]. The most recent metanalysis reports that HBV patients have a two-fold increased probability of death (OR = 2.04, 95% CI 1.49–2.79) if hospitalized for COVID-19 and are more likely to develop severe disease (OR = 1.90, 95% CI 1.32–2.73) [16]. Whether autoimmune hepatitis (AIH) or other autoimmune liver diseases precipitate severe disease from SARS-CoV-2 is still uncertain; however, AIH patients do not seem to have increased rates of COVID-19-related mortality [17].

Another particularity of patients with CLD is the limitations that they may face in standard-of-care treatment for COVID-19. Patients with impaired liver function, defined as elevations of alanine or aspartate aminotransferase (ALT or AST)  ≥ 5× upper limit of normal (ULN) are not to be administered remdesivir, while caution is advised when prescribing tocilizumab due to its hepatoxic potential [18]. Baricinitib, a JAK 1/2 inhibitor, used for its immunomodulating properties on moderate to severe COVID-19, has been described to cause reactivation of HBV [19]. Of note, immunosuppressive treatment for CLD, including chronic hepatitis and AIH is to be continued throughout COVID-19 disease. The initiation of treatment for such a condition is, however, not recommended while sick. The same applies for all autoimmune liver diseases and liver transplant recipients [20].

## 3. CLD-Associated Immunodeficiency

Patients with pre-existing liver pathology are at a noticeably higher risk of COVID-19 infection as well as COVID-19-related death [21]. The main reason behind this is believed to be cirrhosis-associated immune dysfunction (CAID), namely, the pattern of immune dysregulation underlying the reduced functional capacity of the liver tissue, which leads to persistent systemic inflammation and increased susceptibility to bacterial as well as viral infections [22]. Along with endotoxemia, i.e., increased levels of endotoxins in the portal vein, as well as systemic circulation mainly due to increased intestinal permeability, CAID tends to exaggerate immune response in the presence of infection and intensify gut microbiota alterations [23]. Cytokine storm implicated in the pathogenesis of COVID-19 has the potential to inflict further liver damage, that may result in coagulation problems and microthrombi formation [24]. Changes in gut microbiota have also been known to reflect the severity of COVID-19 disease [25].

As far as the function of the immune system in CLD is concerned, the ratio of CD8+/CD4+ effector cells increases as the disease progresses. This increase in CD8+ counts plays an important role in sustaining liver dysfunction and partially participates in the dysregulation of all innate and adaptive immunity cells [26]. Defective and inadequate populations of CD4+ cells are also common features of cirrhosis [27]. B-cell populations are impacted as well, resulting in profound lymphopenia, that may be detectable even in early stages [28]. 

## 4. Vaccination against Viral Pathogens

The overall immune dysfunction that is observed in CLD may lead to hypo-responsiveness to vaccination against communicable diseases [29]. In fact, SARS-CoV-2 antibody titers have been shown to decline relatively quickly in patients with cirrhosis [30]. This may in part be attributed to the reportedly weak T-cell response following vaccination [31]. The reduction in CD27+ memory B cell counts in cirrhosis may also explain vaccines’ reduced efficacy in this group of patients [29]. The decline in immunologic memory is a distinct characteristic of cirrhosis that is as irreversible as the disease that causes it [32]. Therefore, patients with CLD may benefit from vaccination at an earlier stage of their disease since their initial immune response may be more robust at that point. Findings from a hepatitis A (HAV) vaccination study in a cirrhotic population confirm this hypothesis, as people with decompensated disease had a fourfold lower seroconversion rate than their balanced counterparts. The conversion rate minimized as Child–Pugh class increased [33]. The Advisory Committee of Immunization Practices (ACIP) is raising awareness on the need of complete and timely vaccination of CLD patients against HAV and HBV, Influenza, pneumococcus, herpes zoster, tetanus, diphtheria, pertussis, and SARS-CoV-2, as individuals with cirrhosis are at a high risk of decompensation under infection of vaccine-preventable pathogens [34].

Regarding CLD in general, vaccination against viral pathogens has proven safe, although not entirely effective. A recent meta-analysis of 12 studies that measured immunological response following seasonal flu vaccination estimated the seroconversion rate in patients with CLD at 80% for influenza A and at 87% for influenza B, when the recommended lower accepted threshold is at 40%. No difference was noted, however, in all-cause hospitalization or mortality [35]. HBV vaccines also achieve suboptimal humoral immune response that wanes as liver disease severity increases [36,37].

### 4.1. Vaccination against SARS-CoV-2 

Regarding SARS-CoV-2, existing real world data on CLD patients’ vaccination, primarily concern the most widely distributed vaccines, namely the two mRNA vaccines, BNT162b2 (Pfizer-BioNTech) and mRNA-1273 (Moderna, NIAID, BARDA), and the adenovirus vector (AdV) based vaccine Ad26.COV2.S (Janssen Pharmaceutical Companies). A recent study from the U.S. comparing CLD patients to liver transplant recipients demonstrated that 75% of patients with CLD without cirrhosis and 77% of patients with cirrhosis mounted a sufficient antibody response to the mRNA COVID-19 vaccine. In contrast, the Ad26.COV2.S vaccine was associated with poor immunogenicity, as was liver transplant recipient status [38]. Preliminary results on CLD patients from the vaccines’ trials have not been publicly available, nor any significant adverse events concerning the liver were reported [39,40,41]. Regarding other vaccines, mild to moderate increases in total bilirubin and serum alanine aminotransferase levels, in around 8% of all recipients, was reported in Ad5-nCoV-S (CanSino Biologics) vaccine’s preliminary results [42].

Despite the relative lack of quality evidence in vaccine efficacy rates, a retrospective study’s results from the U.S. investigating the efficacy and safety of the two mRNA vaccines in cirrhotic populations reveal a reduction in both hospitalization rates and reported infections for both compensated and decompensated subgroups, even after a single dose administration [43]. Another U.S.-based retrospective study reported no difference in risk of infection or adverse events following vaccination with mRNA formulations within a 30-day period in cirrhotic populations. Hepatic compensation status also did not result in any significant difference [44]. 

Moreover, studies have been carried out to assess the immune reaction and identify potential predictors of low response in CLD. Ruether et.al. [31] compared the humoral and T-cell response of cirrhotic patients and liver transplant recipients following the second vaccine dose. Anti-S protein antibody titers and the spike-specific T-cell response were also assessed. Similar to other authors [45], results suggest that the cirrhotic group, which included patients with decompensated cirrhosis, classified as Child–Pugh class B or C or even after TIPS implantation, had an antibody response similar to healthy controls while almost 50% of liver transplant recipients showed absence or limited spike-specific antibody response. The majority of the LT recipients (78%) did not display any evidence of a spike-specific T-cell response, whereas 56% of cirrhotic patients did. There is strong evidence to suggest that T-cell response may have a protective effect on the patient by minimizing the viral replication even under a lack of a measurable protective antibody titer [46]. Another study reported that CLD patients with cirrhosis, even though they display high immunogenicity upon vaccination, tend to present a rather premature deterioration of their antibody titers compared to healthy controls. Importantly, the decline in titers correlated to the time elapsed from the second dose of the vaccine, suggesting a benefit from an early booster shot administration [30]. In line with these findings, a recent study noted a robust humoral response in patients with cirrhosis, comparable to that of healthy individuals, only after the booster [47]. Results have been replicated in similar studies even involving liver transplant recipients [48]. The role of booster shots becomes even more relevant with the emergence of new variants that may lead to breakthrough infection, especially in immunocompromised individuals [49].

#### 4.1.1 Vaccination-Related Adverse Events

Taking into account that both SARS-CoV-2 vaccine platforms (mRNA and AdV) are newly licensed, vaccination-related adverse events have become a subject of great interest for scientists and common public alike. As stated in the Vaccine Adverse Event Reporting System (VAERS), the most frequent adverse effects concerning the mRNA vaccines are injection site pain (70.9%), fatigue (33.5%), and headache (29.5%), where for the J&J vaccine, they were, additionally to the aforementioned, myalgia (47.8%), fever (34.7%), and chills (34.2%) [50]. Anaphylactic reactions have also been reported after both vaccination formulation with the overall incidence of anaphylaxis for the mRNA vaccine being 4.5 cases per million doses administered. This rate is comparable to the one reported on other commonly administered vaccines, such as the inactivated influenza vaccine, the pneumococcal vaccine, and live attenuated herpes zoster vaccine [51], whereas for the Ad26.COV2.S vaccine the same rate stands at lower than 0.5 cases per million. Such cases of immediate allergic reactions were dealt with common measures and did not represent life-threatening events, despite that they resulted in emergency room visits in 12.5% of cases [52]. Rarely reported immune-mediated delayed reactions including myocarditis, thrombosis with thrombocytopenia and Guillain Barre Syndrome, remain uncommon and certainly do not exceed the risk of severe outcomes following COVID-19 infection [53].

Evidence in patients with CLD and vaccine associated adverse events comes from a recent metanalysis including five studies (inactivated vaccine—4, mRNA vaccine—1) reporting adverse events with 1360 subjects [45]. Prevalence of adverse events was 27% (95% CI, 18–38%), although significant heterogeneity was observed. Three subjects were reported to have laboratory abnormalities with raised alanine transaminase five times above upper limit of normal, two subjects presented severe systemic side effects and required medications, while two required hospitalizations [31,54]. The most common local and systemic adverse event was pain (13%) followed by fatigue (3%) [55]. Interestingly, pooled prevalence of adverse events in LT recipients was 63% [45,56]. In terms of serious adverse events, following vaccination in patients with cirrhosis and a history of decompensation, an incidence of up to 1.2% has been noted [57]. 

Rare incidents of immune-mediated hepatitis and liver injury have also been reported following SARS-CoV-2 vaccination. The largest case series to date highlights the rarity of such events with only 84 cases identified across 14 countries and points to the fact that most of them are in fact immune-mediated [58]. The pattern of injury was mostly hepatocellular and the majority of patients presented with symptoms, including jaundice in 39% of them. Only about 15% of the cases had a history of liver disease. Mean time of presentation was 15 days following vaccination and almost half of the cases were diagnosed after the second vaccine dose. Incidence was noted to be higher in the group of individuals vaccinated with the BNT162b2 vaccine; however, the difference is attributed to local vaccine availability and distribution. Almost half of the patients required immunosuppressive therapy in the form of corticosteroid administration, and eventually only one required liver transplant [58]. Concerning immune-mediated injury in particular, a review assessing most reported cases, identified pre-existing liver disease in about 25% of them. All of the patients recovered, with 86% of them requiring immunosuppressive therapy [59]. The most conclusive study concerning liver injury, conducted in Hong Kong was published recently and reported no increased risk following vaccination with the mRNA and inactivated vaccines [60].

## 5. Current Recommendations

At the moment, vaccination and boosters are strongly recommended in patients with CLD and LT recipients according to recent American Association for the study of Liver Diseases (AASLD) and European Society for Liver Diseases (EASL) guidelines schedule [61,62]. As the severity of the liver disease progresses and time since last vaccination elapses, protection against SARS-CoV-2 and its variants declines; therefore, vaccines and booster shots should be administered as timely as possible. Nonetheless, all patients with CLD and LT recipients, including vaccine recipients, should continue to mitigate their risk of SARS-CoV-2 exposure e.g., masking, social distancing, hand washing, etc. Serological testing for evidence of prior SARS-CoV-2 infection is not recommended prior or after COVID-19 vaccination. When liver biochemistries increase following vaccination and a delay to return to baseline is noted, a thorough evaluation for other causes should be performed. Reports of AIH or other rare adverse events should not hamper vaccination efforts. Vaccination with other vaccines such as influenza, pneumococcus, and other inactivated vaccines should continue as normal. Notably, the latter can be administered at the same time as COVID-19 vaccines.

However, when surveyed, although more than 85% of participants with various kinds of chronic liver or gastrointestinal conditions agreed on the safety of vaccination and even stated that they would take the COVID-19 vaccine if available, about 8% completely disagreed and concerns about vaccination were expressed on 33% of participants. Female sex, black race, and lower household income by zip code were associated with higher hesitancy [63].

## 6. Unresolved Issues and Future Directions

CLD includes a wide spectrum of conditions each with a distinct and highly variable underlying immune profile. Currently available vaccines have provided us with a useful tool for CLD patients to deal with COVID-19 pandemic; however, a number of issues are still of concern. Knowledge gaps remain as to effectiveness, safety, and durability in patients with CLD based on liver disease etiology, comorbidities, Child–Turcotte–Pugh class, and Model for End-Stage Live Disease score, reflecting different amount of immunosuppression. Novel vaccine platforms have not yet been thoroughly tested in all CLD subgroups with variable degrees of immunocompromise also depending on underlying medication. Similarly, difference in vaccine effectiveness, safety, and optimal timing is yet to be elucidated in patients previously infected with SARS-CoV-2, even though booster shots are clearly recommended even in this subgroup of patients. Data are—and are condemned to be—limited to currently circulating variants of concern. In the context of an evolving pandemic, available information is to be constantly reassessed in order to optimize best practice and also define long-term COVID-19 effects in patients with CLD. In the setting of an already diverse population including different racial and ethnic backgrounds, experience in CLD patients with need of vaccine platform combination following adverse events or failure is lacking, although partially studied in the general population. Determination of antibody cut-offs, interpretation of serology and cell-mediated immunity assays are pivotal, but still ambiguous in these patients in the setting of their comorbidities. We are looking forward to the results of several randomized i.e., NCT04775069, NCT04794946, and observational trials i.e., NCT04798625, NCT04775056, testing immunogenicity and safety exclusively in this population and providing more insight to unresolved matters of concern [64].

## 7. Conclusions

Patients with CLD carried a significant burden of morbidity and mortality during COVID-19 pandemic. Experience with currently available vaccines is encouraging in these patients, confirming adequate immunogenicity and good safely profile, in line with results in general population. Nonetheless, a number of issues are still to be elucidated in this diverse—in terms of etiology and disease severity—population. Liver specialists, except for infectious diseases practitioners attending patients with CLD should embrace and advocate the importance of adequate and timely vaccination, as a method of eliminating pandemic resurgence and protecting this most vulnerable patient group. Increase of patient awareness and education remains pivotal.

## Data Availability

Not applicable.
